# Association of dietary pattern and body weight with blood pressure in Jiangsu Province, China

**DOI:** 10.1186/1471-2458-14-948

**Published:** 2014-09-12

**Authors:** Yu Qin, Alida Melse-Boonstra, Xiaoqun Pan, Jinkou Zhao, Baojun Yuan, Yue Dai, Minghao Zhou, Johanna M Geleijnse, Frans J Kok, Zumin Shi

**Affiliations:** Department of Non-communicable Chronic Disease Control, Jiangsu Provincial Center for Disease Control and Prevention, Nanjing, P. R. China; Division of Human Nutrition, Wageningen University, P.O. Box 8129, 6700 EV, Bomenweg 2, Bdg 307, 6703 HD Wageningen, The Netherlands; Discipline of Medicine, University of Adelaide, Adelaide, Australia

**Keywords:** Dietary pattern, Body weight, Salt, Blood pressure, China

## Abstract

**Background:**

To identify risk factors, associations between dietary patterns, body mass index (BMI), and hypertension in a Chinese population.

**Methods:**

Dietary intake was assessed in 2518 adults by a 3-day 24 h recall and a food frequency questionnaire. Salt and oil intake was assessed by weighing records. Four dietary patterns were identified using principal component analysis. Overweight and obesity was determined according to the Chinese cut-offs for BMI. High blood pressure was defined as systolic blood pressure ≥ 140 mmHg and/or diastolic blood pressure ≥ 90 mmHg. Prevalence ratios (PR) were calculated using Poisson regression.

**Results:**

Of the subjects, 26.7% had high blood pressure. Subjects with overweight and obesity were more likely to have high blood pressure than those with normal weight (PR, 95% CI: 1.60, 1.40-1.87; 2.45, 2.11-2.85, respectively). Subjects with a ‘traditional’ dietary pattern were more likely to have high blood pressure (P _for trend_ = 0.001), whereas those with a ‘macho’ or ‘sweet tooth’ dietary pattern were less likely to have high blood pressure (P _for trend_ = 0.004 and <0.001, respectively). More than half of the population had salt intakes > 9 g/d, and blood pressure increased with salt intake (P _for trend_ <0.001). Subjects with a ‘traditional’ dietary pattern had the highest salt intake (12.3 g/d).

**Conclusion:**

A traditional dietary pattern is associated with high blood pressure among the population of Jiangsu Province, which may be mainly due to high salt intake. Moreover, high BMI is an important determinant of high blood pressure. Both issues need to be addressed by lifestyle interventions.

## Background

Hypertension has been identified to be the first leading risk factor of mortality and the third leading risk factor of the total burden of disease globally [1]. It was estimated that a quarter of the world’s adults had hypertension in 2002, and that the proportion will increase to 29% by 2025 [2]. Hypertension contributes to premature death and disability from cardiovascular diseases and stroke; peripheral vascular disease; and kidney failure [3]. In China, the prevalence of hypertension in the adult population has quadrupled from 5% in 1959 to nearly 19% in 2002, which comprises approximately 200 million people [4]. In addition, awareness of hypertension is poor. According to data from the China National Nutrition and Health Survey of 2002, less than one quarter of the hypertensive population are aware of having hypertension, and only one quarter is adequately treated and controlled [5].

Many studies have indicated that body mass index (BMI) is generally positively associated with blood pressure [6], which is also the case in China [7]. Overweight and obesity is an emerging epidemic in China, with almost 30% of the adult population being classified as either overweight or obese [8]. The high prevalence of hypertension as well as of overweight and obesity in China can be attributed to the recent economic development and urbanization accompanied by unfavorable changes in diet and lifestyle. The prevalence of hypertension is known to be higher in the north as compared to the south of China, which has been attributed both to dietary factors [9], and to the higher prevalence of overweight and obesity in northern China [10].

Multiple dietary factors affect blood pressure. A large-scale intervention study on Dietary Approaches to Stop Hypertension (DASH) revealed that a dietary pattern rich in fruits and vegetables, rich in low-fat dairy products, and reduced in saturated fat and cholesterol reduces the risk of hypertension [11]. Furthermore, dietary sodium reduction, regular aerobic physical activity, and moderation of alcohol consumption will help to maintain normal blood pressure [12]. Hypertension has been reported in relation to some western and South-East Asian dietary patterns [13–15]. Data from the Shanghai Men’s Health Study showed that a dietary pattern consisting mainly of fruit and milk was inversely associated with blood pressure among middle-aged and elderly men [16]. Wang et al. [10] found that a typical traditional southern Chinese dietary pattern, characterized by high intakes of fruit, pork, poultry, rice, vegetables, aquatic products and nuts, was inversely related with hypertension independent of BMI [10].

Jiangsu Province, located at the mid-east coast of China, is an economically booming area with a population of 73.6 million. Dietary and lifestyle habits have changed dramatically in this Province over the past two decades [17]. The current age-standardized prevalence of the metabolic syndrome has been estimated to be 30.5%, with high blood pressure as the leading component (45.2%) among its population [18]. However, the etiology of hypertension in this Province is so far unexplained. Therefore, we aimed to investigate the associations between dietary patterns, BMI, and hypertension in a representative sample of the population of Jiangsu Province.

## Methods

### Subjects

The study was conducted in Jiangsu Province using a multistage cluster sampling method, as described previously [19, 20], which was part of the 2002 National representative cross-sectional survey in nutrition and health. Six counties and two prefectures represented a geographically and economically diverse population for Jiangsu Province. From each of the six areas, three streets/towns were randomly selected. In each street/town, two villages/neighbourhoods were further randomly selected. In each village/neighbourhood, thirty households were randomly selected. All members in the households were invited to take part in the study, and adults aged 20 years and above were included in our study. Those already diagnosed with hypertension, diabetes, dyslipidemia, stroke and cardiovascular diseases were excluded from the study, because they may have changed their dietary habits. Among 2832 subjects, 311 (11%) had already been diagnosed with hypertension. In total, 2518 subjects with 1146 males and 1372 females were included in the data analysis. Written consents were obtained from all the participants. The study was approved by the Human Investigation Review Committee at the National Institute for Nutrition and Food Safety, Chinese Center for Disease Control and Prevention.

### Dietary intake measurement

Trained interviewers from the local Center for Disease Control and Prevention visited subjects in their homes to collect information on food intake using a 24-h dietary recall method on three consecutive days, including two weekdays and one weekend day. Energy and nutrient intake was calculated by SAS Software using the dietary recall data in conjunction with the China Food Composition Tables published in 2002 [21].

Salt, oil, and condiments which contributed to salt intake, such as soy sauce, vinegar, and monosodium glutamate, were weighed at the 1^st^ 24-h recall, and again at the 2^nd^ recall 24 h later. The household salt and oil consumption was calculated as the difference between the two weighings. Individual salt and oil intake was estimated based on the proportion of each household member’s food consumption, and categorized into quartiles.

### Dietary patterns

A validated food frequency questionnaire (FFQ) was used to collect dietary information over the past year [22]. The FFQ included a series of detailed questions regarding the usual frequency and quantity of intake of thirty-three foods and beverages. This was further merged into twenty-five food items in the analysis because of the low intake of some food items. Portion size for each food was established by using food models. Subjects were asked to recall the frequency of consumption of individual food items (number of times per day, per week, per month, per year) and the estimated portion size, using local weight units (1 liang =50 g) or natural units (cups). Intakes of foods were converted into g/week for data analysis. Use of vitamin and mineral supplements was included in the questionnaire, but because these were very seldomly used in the area, they were not included in this analysis.

Dietary patterns were identified by factor analysis, using standard principal component analysis as described before for this population [19]. Four different patterns were defined: 1) the ‘traditional’ pattern (characterized primarily by consumption of rice and freshly cooked vegetables, secondary of pork and fish, and lastly of root vegetable and wheat flour); 2) the ‘macho’ pattern (characterized primarily by consumption of animal foods and alcohol, and secondary of eggs, fish, nuts, and fruits); 3) the ‘sweet tooth’ pattern (characterized primarily by consumption of cake, milk, yoghurt and drinks, secondary of animal foods, nuts and fruits, and lastly of pickled vegetables and alcohol); and finally 4) the ‘healthy’ pattern (characterized primarily by consumption of whole grains, fruits, pickled vegetables, and secondary by fresh vegetables, milk, eggs and fish). The four factors explained 30.5% of the total variance in intake (10.6%, 8.6%, 5.9% and 5.4% for ‘traditional’ , ‘macho’, ‘sweet tooth’ , and ‘healthy’ patterns, respectively). Scores for each pattern were calculated as the sum of the products of the factor loading coefficient and the standardized weekly intake of each food associated with that pattern. Only foods with factor loadings of more than 0.20 and less than -0.20 were included in calculation of pattern scores because these items represent the foods most strongly related to the identified factor. Factor scores were divided into quartiles. The scores (intakes) increased from quartile 1 (Q1) to quartile 4 (Q4).

### Anthropometric measurement

Weight was measured in light indoor clothing without shoes to the nearest 10th of a kilogram. Height was measured without shoes to the nearest 10th of a centimeter with a stadiometer. Waist circumference was measured at 1 cm above the navel at minimal respiration. All measurements were performed twice during the visit by trained observers using a standard protocol and techniques [23]. Body mass index (BMI) was calculated as weight in kilograms divided by height in squared meters. Subjects were classified into BMI categories as underweight (BMI < 18.5), normal weight (BMI > 18.5 < 24), overweight (BMI ≥ 24 < 28) and obese (BMI ≥ 28) according to Chinese standards [24].

### Blood pressure measurement

Blood pressure was measured twice on the right arm by trained investigators with the participants in a seated position after 5 minutes of rest, using a standard mercury sphygmomanometer and appropriate-sized cuff according to a standard protocol [25]. The mean of those two measurements was used for analyses, with a coefficient of variation of 1.28% and 1.78% for systolic and diastolic blood pressure, respectively. High blood pressure was defined as systolic blood pressure ≥ 140 mmHg and/or diastolic blood pressure ≥ 90 mmHg.

### Physical activity

Information on physical activity was collected using a validated physical activity questionnaire covering a time period of one year [26]. Questions on daily commuting to and from work were categorized into three categories: (1) using motorized transportation or not (0 min of walking or cycling); (2) walking or bicycling 1–29 min; (3) walking or bicycling for >30 min. Daily leisure-time physical activity including boxing, running, walking, etc., was classified into 0; 1–29; ≥ 30 min.

### Alcohol use and socio-economic status

Alcohol use was assessed by asking the participants about the frequency and amount of alcohol/beer intake, and categorized into three categories, namely <0, 0–9, and ≥ 10 g/d. Low socio-economic status (SES) was defined as an income of less than 1999 Yuan, ‘medium’ as 2000–4999 Yuan and ‘high’ as more than 5000 Yuan.

### Statistical analysis

The food intakes followed normal distribution. Variables were presented as percentage or mean ± standard deviation (SD). Student’s *t*-test, ANOVA and chi-square test was used to determine subgroup differences for continuous and qualitative variables, respectively. Poisson analysis was performed using SAS 9.2 to analyze associations between BMI, salt intake, dietary pattern and high blood pressure with household, age, gender and other known risk factors including SES, salt and potassium intake, physical activity and alcohol use as confounders. All other analyses were performed using SPSS 19.0 (IBM SPSS Inc., USA). Statistical significance was set at α = 0.05.

## Results

The mean age of the subjects was 47.0 ± 14.5 years old, and 26.7% had high blood pressure. Older subjects had a higher prevalence of high blood pressure than younger subjects (P _for trend_ <0.001). The prevalence reduced in subjects with more active commuting activities (P _for trend_ <0.001), and increased with more leisure time activities (P _for trend_ <0.001). Compared with never drinkers, alcohol drinkers had a higher prevalence of high blood pressure (P _for trend_ <0.001). The prevalence of hypertension increased with salt intake (P _for trend_ <0.001) and over BMI categories (P _for trend_ <0.001). No differences were found for gender, SES and potassium intake (Table 1).

**Table 1:** **Subject characteristics by blood pressure status** ^**1**^

	Blood pressure ^2^		P
	Normal	High	
**N**	1845	673	
**gender**			
Male	831 (45.0)	315 (46.8)	0.43
Female	1014 (55.0)	358 (53.2)	
**Age**			
20-29	270 (14.6)	10 (1.5)	< 0.001
30-39	459 (24.9)	51 (7.6)	
40-49	423 (22.9)	124 (18.4)	
50-59	292 (15.8)	191 (28.4)	
60+	401 (21.7)	297 (44.1)	
**SES**			
Low	570 (31.2)	216 (32.3)	0.31
Middle	583 (31.9)	225 (33.6)	
High	672 (36.8)	228 (34.1)	
**Active commuting**			
None	628 (34.0)	325 (48.3)	< 0.001
1-30 min/d	966 (52.4)	268 (39.8)	
>30 min/d	251 (13.6)	80 (11.9)	
**Leisure time activity**			
None	1704 (92.4)	577 (85.7)	< 0.001
1-30 min/d	70 (3.8)	43 (6.4)	
>30 min/d	71 (3.8)	53 (7.9)	
**Alcohol drinking**			
Never	1590 (86.2)	552 (82.0)	0.005
Low	142 (7.7)	61 (9.1)	
High	113 (6.1)	60 (8.9)	
**Salt intake**			
< 6	474 (25.7)	154 (22.9)	< 0.003
6-9	428 (23.2)	140 (20.8)	
9-14	523 (28.3)	178 (26.4)	
≥14	420 (22.8)	201 (29.9)	
**Potassium intake**			
< 1.28	478 (25.9)	162 (24.1)	0.57
1.28-1.56	463 (25.1)	163 (24.2)	
1.56-1.94	437 (23.7)	189 (28.1)	
≥1.94	467 (25.3)	159 (23.6)	
**BMI**			
<18.5	110 (6.0)	18 (2.7)	<0.001
18.5-24	1134 (61.5)	266 (39.5)	
24-28	485 (26.3)	238 (35.4)	
≥28	116 (6.3)	151 (22.4)	

^1^Data are presented as N (%). Differences between groups were analyzed by chi-square test. ^2^High blood pressure is defined as SBP ≥ 140 mmHg and/or DBP ≤ 90 mmHg.

The prevalence of overweight and obesity was 28.8% and 10.6% among subjects, respectively. Subjects with overweight and obesity were more likely to have high blood pressure than those with normal weight (PR: 1.60, 95% CI: 1.40-1.87; PR: 2.45, 95% CI: 2.11-2.85, respectively), after adjustment for household, age, gender, SES, salt and potassium intake, physical activity and alcohol use (Table 2).

**Table 2:** **Prevalence ratios (95% CI) of high blood pressure among BMI categories**

BMI	Model 1	Model 2	Model 3
**Underweight**	0.74 (0.48-1.15)	0.68 (0.44-1.03)	0.71 (0.46-1.08)
**Normal**	1	1	1
**Overweight**	1.73 (1.49-2.01)	1.60 (1.38-1.85)	1.60 (1.40-1.87)
**Obesity**	2.98 (2.56-3.46)	2.46 (2.12-2.85)	2.45 (2.11-2.85)
**P for trend**	<0.001	<0.001	<0.001

Model 1 crude model.

Model 2 adjusted by household, age and gender.

Model 3 additionally adjusted by SES, salt intake, potassium intake, alcohol use and physical activity.

The average salt intake was 11.4 ± 9.6 g/d, and there was a positive association between salt intake and high blood pressure (P _for trend_ < 0.01), independent of household, age, gender, SES, potassium intake, physical activity, BMI and alcohol use (Table 3).

**Table 3:** **Prevalence ratios (95% CI) of high blood pressure among salt intake categories**

Salt intake (g/d)	Model 1	Model 2	Model 3
< 6	1	1	1
6-9	1.00 (0.82-1.23)	1.01 (0.84-1.22)	0.98 (0.81-1.17)
9-14	1.03 (0.86-1.25)	1.06 (0.89-1.27)	1.00 (0.84-1.18)
≥14	1.32 (1.10-1.58)	1.32 (1.10-1.56)	1.21 (1.02-1.43)
**P for trend**	0.003	0.003	0.01

Model 1 crude model.

Model 2 adjusted by household, age and gender.

Model 3 additional adjusted by SES, BMI, potassium intake, alcohol use and physical activity.

Salt intake increased over quartiles of the ‘traditional’ pattern, and decreased over quartiles of the ‘sweet tooth’ pattern. Fresh vegetable intake was highest in the highest quartile of the ‘traditional’ pattern. Potassium and energy intake increased over quartiles of the ‘traditional’ , ‘Macho’ and ‘healthy’ patterns, and decreased over the ‘sweet tooth’ pattern (Table 4).

**Table 4:** **Food and nutrient intakes related to high blood pressure in the lowest and highest quartiles of dietary patterns**

	‘Traditional’		‘Macho’		‘Sweet tooth’		‘Healthy’	
	Q1	Q4	Q1	Q4	Q1	Q4	Q1	Q4
**Salt (g/d)**	11.4 ± 8.9	12.3 ± 11.9*	11.2 ± 9.2	11.3 ± 9.1	13.7 ± 11.8	9.4 ± 6.6*	10.9 ± 9.1	11.5 ± 9.5
**Meat (g/d)**	42.9 ± 57.9	113.7 ± 89.2*	54.0 ± 62.8	119.9 ± 93.0*	74.9 ± 86.5	110.3 ± 81.9*	103.1 ± 79.4	85.1 ± 87.2*
**Fresh vegetable (g/d)**	256.5 ± 173.6	326.7 ± 144.5*	288.4 ± 177.2	282.8 ± 158.7	302.9 ± 167.3	251.4 ± 127.6*	285.1 ± 142.5	285.9 ± 169.6
**Oil (g/d)**	44.7 ± 29.1	43.7 ± 28.5*	40.7 ± 28.7	42.5 ± 27.5	48.2 ± 30.4	38.2 ± 23.2*	37.6 ± 27.1	44.8 ± 28.7*
**K (g/d)**	1.8 ± 0.6	1.8 ± 0.7*	1.6 ± 0.6	1.7 ± 0.6*	1.8 ± 0.6	1.6 ± 0.5*	1.6 ± 0.6	1.8 ± 0.7*
**Energy (KJ/d)**	2510.2 ± 734.9	2505.7 ± 669.6*	2306.0 ± 675.0	2476.8 ± 718.1*	2677.3 ± 688.5	2111.7 ± 599.1*	2273.5 ± 654.5	2465.8 ± 731.5*

*P < 0.05 over quartiles analyzed by ANOVA.

Table 5 shows that the prevalence of high blood pressure increased over quartiles of the traditional dietary pattern (P _for linear trend_ = 0.006), and decreased over quartiles of the Macho dietary pattern (P _for linear trend_ = 0.02) and the ‘sweet tooth’ dietary pattern (P _for linear trend_ <0.001). There was no trend in the prevalence over quartiles of the healthy dietary pattern. The trends remained similar after adjustment for household, age, gender, SES, physical activity, BMI and energy intake.

**Table 5:** **Prevalence ratios (PRs) of high blood pressure among dietary patterns**

Dietary pattern	%	Model 1	Model 2	Model 3
**‘Traditional’**				
**Q1**	21.1	1	1	1
**Q2**	28.2	1.34 (1.09-1.65)	1.37 (1.10-1.71)	1.30 (1.05-1.61)
**Q3**	28.0	1.33 (1.09-1.62)	1.37 (1.10-1.71)	1.51 (1.21-1.88)
**Q4**	28.7	1.36 (1.11-1.66)	1.39 (1.12-1.71)	1.47 (1.18-1.82)
**P for trend**		0.006	0.007	0.001
**‘Macho’**				
**Q1**	29.9	1	1	1
**Q2**	27.0	0.90 (0.76-1.08)	0.90 (0.76-1.07)	0.92 (0.77-1.09)
**Q3**	25.8	0.86 (0.72-1.03)	0.85 (0.71-1.02)	0.82 (0.69-0.98)
**Q4**	24.1	0.81 (0.67-0.97)	0.80 (0.66-0.96)	0.78 (0.65-0.94)
**P for trend**		0.02	0.02	0.004
**‘Sweet tooth’**				
**Q1**	31.8	1		
**Q2**	27.5	0.86 (0.73-1.02)	0.84 (0.71-1.00)	0.86 (0.73-1.02)
**Q3**	24.0	0.76 (0.63-0.91)	0.72 (0.60-0.86)	0.75 (0.62-0.90)
**Q4**	23.4	0.74 (0.61-0.89)	0.67 (0.55-0.82)	0.71 (0.58-0.86)
**P for trend**		0.0004	< 0.0001	0.0001
**‘Healthy’**				
**Q1**	26.5	1	1	1
**Q2**	26.1	0.98 (0.82-1.18)	0.99 (0.82-1.19)	0.92 (0.77-1.10)
**Q3**	25.7	0.97 (0.80-1.17)	0.97 (0.81-1.17)	0.88 (0.74-1.06)
**Q4**	28.6	1.08 (0.90-1.29)	1.09 (0.91-1.30)	0.89 (0.74-1.07)
**P for trend**		0.45	0.19	0.19

Q1 is the lowest quartile, Q4 is the highest quartile.

Model 1 crude model.

Model 2 adjusted by household, age and gender.

Model 3 additionally adjusted by SES, BMI, physical activity and energy intake.

## Discussion

We found that the ‘traditional’ dietary pattern was positively associated with hypertension, whereas the two more westernized dietary patterns showed a negative association. BMI and salt intake both were strong determinants of hypertension, but also age, and alcohol intake were associated with high blood pressure, whereas physical activity.

A main strength of the study is that the sampling is based on a representative population including different sociodemography and geography. We used dietary weighing in combination with a 3-day 24-h recall in the study which provided a relatively accurate estimation of salt intake. As a cross-sectional study, the main limitation is that we cannot establish a causal relationship between dietary patterns and high blood pressure. However, we excluded subjects with diagnosed hypertension and related diseases to avoid possible dietary change following clinician’s suggestions and thereby maintaining a natural association between dietary intake and blood pressure in the study population. Misclassification of food intake may have occurred, although we used a validated food frequency questionnaire in the survey. The salt intake is estimated based on household salt use, which may underestimate real intake. Measuring 24 urine sodium excretion would be a better choice, however, it would be a challenge to obtain complete 24 hour urine collections from a large population and it would still be hard to quantify habitual intake because of day-to-day variation [27]. In our study we used 24-h recall combination with dietary weighing, which is better than FFQ only.

Our study shows that the prevalence of high blood pressure was positively associated with the ‘traditional’ dietary pattern. This is counterintuitive, since the ‘traditional’ dietary pattern, with its abundant amount of fresh vegetables, is more in line with the DASH diet than any of the other dietary patterns in our population. Effective prevention and treatment of hypertension has been shown for the Mediterranean Diet, as a model of DASH, with abundant amounts of fresh vegetable intake usually in the form of salads without cooking [13]. However, eating raw vegetables is not common in many other parts of the world. For example, in Korea, subjects with a traditional dietary pattern mostly consumed salted vegetables resulting in high sodium intake [15]. Moreover, Chinese vegetarian diets contain large amounts of salt since these are predominantly based on soy products with little taste, which differs from western vegetarian diets. Therefore, Chinese vegetarian diets tend to have high sodium content, which may predispose to hypertension [28]. In Jiangsu Province, vegetables are traditionally cooked and then stir-fried with a large amount of oil and salt, which may contribute to high energy and sodium intake. We also found a clear association between salt intake and hypertension, and salt intake was highest in subjects with a traditional dietary pattern. Therefore, our results may be explained by food preparation habits related to the traditional dietary pattern. Our findings are consistent with several other studies in Asian populations that found a positive association between a traditional dietary pattern rich in vegetables and hypertension [10, 15, 16]. However, the opposite has also been reported with a negative association between a traditional dietary pattern and hypertension in southern China, which included fruit, poultry, pork, aquatic product, soybean product and vegetables [10], suggesting that factors other than vegetable intake play an important role.

Salt intake has been acknowledged as a direct risk factor for hypertension [29]. Salt (sodium chloride) is distributed predominantly to the extracellular space. The rise in extracellular volume by excess amounts of salt intake results in increased cardiac output and rising blood pressure [30]. A meta-analysis of 17 randomized trials showed that modest and long-term reduction of salt intake lowers blood pressure in both hypertensive and normotensive individuals [31]. In addition, dietary sodium reduction is related to decreased blood pressure [32, 33] and reduces the risk of cardiovascular outcomes by 25-30% [34]. WHO recommends a salt intake of no more than 5 g per day [35], however, the majority of our study population consumed salt at amounts much higher than this recommendation. The inverse association between ‘sweet tooth’ pattern and hypertension that we found may be due to the relatively low intake of salt in combination with low vegetable and oil intake.

In our study, overweight and obese subjects were more likely to have high blood pressure. Maintaining a normal body weight (BMI 18.5-24.9 kg/m^2^) is recommended for prevention and management of hypertension [12]. Two meta-analyses of randomized controlled trials showed that weight loss contributed to a reduction in both systolic and diastolic blood pressure [6, 36]. It has been estimated that weight loss of 10 kg can reduce 5–20 mmHg of systolic blood pressure [12]. Obesity is associated with hyperleptinemia via secreting several immunomodulators and bioactive molecules by adipose tissue [37, 38]. Leptin, which helps blood volume and pressure homeostasis in normal conditions, increases blood pressure through activation of the sympathetic nervous system during chronic hyperleptinemia [38, 39].

We found a negative association between the more westernized ‘sweet tooth’ dietary pattern and the prevalence of hypertension in Jiangsu Province. In contrast, a clear positive association between a ‘western’ dietary pattern and hypertension was found on the national level in China [10]. As compared to the ‘western’ dietary pattern in the national study, we found that subjects with a predominant ‘sweet tooth’ dietary pattern had lower salt intake than for example those with a ‘traditional dietary pattern’ , which may partly explain our findings. Moreover, we previously found a negative association between the ‘sweet tooth’ dietary pattern and central obesity [40]. It may be that the nutrition and epidemiologic transition at the time of the study had only just begun in Jiangsu Province thereby not yet showing an association between exposure and disease outcomes, whereas in other parts of China this has begun earlier. Analysis of follow up data will throw more light on this.

The ‘Macho’ pattern with a high intake of meat also showed negative association with blood pressure, which is inconsistent with some other reports [41–43]. A cross-sectional study showed that meat eaters had a higher prevalence of hypertension than non-meat eaters, especially vegans [41]. An international collaborative cross-sectional study found that a high intake of red meat with 103 g/d resulted in both higher systolic and diastolic blood pressure [42]. A 10-year follow-up study indicated that red meat intake, but not poultry, was positively associated with the risk of hypertension, compared to those who consumed no red meat [43]. Meat products, particularly red meat, are a major source of saturated fat, animal protein, and cholesterol, which may contribute to the development of hypertension [44]. However, other studies reported that a higher intake of meat lowers blood pressure [45, 46]. The effect of animal meat therefore remains uncertain [47].

## Conclusion

In conclusion, we found that a ‘traditional’ dietary pattern is associated with high blood pressure in Jiangsu Province, which may mainly be due to a higher intake of salt. Overweight and obesity were also directly associated with blood pressure in the population. Our results suggest that decreasing the use of salt and other salt-containing condiments in food preparation should be included in the dietary recommendations for the prevention of high blood pressure. Our findings may be generalized to other parts of China and other Asian countries with similar cooking habits, although dietary patterns may differ. Public health measures including mass education campaigns with dietary recommendations should be conducted to promote healthy lifestyles including weight management and reduction of salt use in China. Further studies should be conducted to examine the association between blood pressure and salt intake in this population more closely.

## Abbreviations

BMI: Body mass index
DASH: Dietary Approaches to Stop Hypertension
FFQ: Food frequency questionnaire
PR: Prevalence ratios
SES: Socio-economic status
SD: Standard deviation.
